# Coronary atherosclerosis screening in asymptomatic adults using coronary artery calcium for cardiovascular prevention: a systematic review of randomised controlled trials and prospective cohorts

**DOI:** 10.1136/bmjopen-2025-101472

**Published:** 2025-07-05

**Authors:** Victor Scheu, Louhai Alwan, Christoph Gräni, Baris Gencer, Nicolas Rodondi, Manuel R Blum

**Affiliations:** 1 Department of General Internal Medicine, Inselspital University Hospital Bern, Bern, Switzerland; 2 Institute of Primary Health Care (BIHAM), University of Bern, Bern, Switzerland; 3 Department of Cardiology, Inselspital University Hospital Bern, Bern, Switzerland; 4 Service de Cardiology, Lausanne University Hospital, Lausanne, Switzerland; 5 Department of Medicine, Hospitals Frutigen Meiringen Interlaken, Interlaken, Switzerland

**Keywords:** Primary Prevention, Systematic Review, Coronary heart disease, Computed tomography

## Abstract

**Abstract:**

**Objectives:**

To review the available evidence of screening for atherosclerosis in adults in a primary prevention setting with coronary artery calcium scoring (CACS) on the impact on cardiovascular (CV) risk factor control, health behaviour and clinical events.

**Design:**

Systematic review, reported in accordance with Preferred Reporting Items for Systematic Reviews and Meta-Analyses guidelines.

**Data sources:**

We searched MEDLINE, Embase and Cochrane Central Register of Controlled Trials through 22 January 2025.

**Eligibility criteria:**

We included randomised controlled trials (RCTs) and prospective cohorts, without language restrictions, comparing adults without cardiovascular diseases undergoing CACS to a control group that either did not undergo CACS or where the participants and physicians were blinded to its result. Outcomes included changes in CV risk factor control, CV therapy, changes in health behaviour at follow-up and clinical events (all-cause and CV mortality and non-fatal CV events).

**Data extraction and synthesis:**

Two independent reviewers extracted data and assessed the risk of bias. Due to substantial heterogeneity among the included studies, a quantitative analysis was not possible.

**Results:**

We identified seven RCTs and one observational study, with participants ranging from 56 to 43 447 with a total of 51 554. Populations were heterogeneous with a mean age range of 42–64 years, % women ranging from 21% to 100% and mean baseline CACS from 1.37 to >100 Agatston units. Interventions following CACS were also heterogeneous, ranging from simply communicating results to participants to initiating statin therapy for detectable CACS. One RCT demonstrated improvement regarding blood pressure (BP) (n=2137; change in systolic BP: CACS: −5 mm Hg; control: −7 mm Hg; p=0.02), several an improvement in blood lipids between groups (five studies, n=3693; eg, low-density lipoprotein (LDL) cholesterol: range −6.0 to −4.9 mg/dL). Results regarding CV medication (seven studies, n=51 104) were more discrepant, with some studies showing a decrease and others an increase in indication for or usage of CV medication. Three trials (n=3338) investigated adherence to CV medication, with only one showing increased adherence to statins (CACS: 63.3%; control: 45.6%; p=0.03). Five trials (n=3692) investigated behavioural changes, with one showing an increased motivation to change lifestyle (CACS: 94%; control: 62.8%; p=0.002) and another a higher adherence in self-reported physical activity (CACS: 96%; control: 59%; p<0.01). Due to low event rates, short follow-up and/or limited sample size, none (three studies, n=6552) demonstrated an effect on clinical CV events or all-cause mortality. Heterogeneity in interventions following CACS, population and studied outcomes did not permit pooling of results. Key limitations of this review reflect the limited availability of evidence and include the omission of potential harms of CACS screening, study heterogeneity, insufficient data on clinical events, a lack of economic assessments and the moderate to high risk of bias in most studies.

**Conclusions:**

CACS screening with a CACS-guided intervention might have a favourable effect on CV risk factor control and potentially on adherence to CV medication and increased motivation to change lifestyle in populations at intermediate to high risk. The available evidence is insufficient to determine whether screening asymptomatic patients with CACS has an impact on all-cause mortality or CV events. Despite its known strengths in predicting outcomes in individual patients, more evidence regarding the impact on clinical outcomes is needed to determine the clinical use of CACS for screening purposes in asymptomatic patients.

**PROSPERO registration number:**

CRD42022377727.

**STRENGTHS AND LIMITATIONS OF THIS STUDY**

- A systematic review focusing on the screening using coronary artery calcium scoring (CACS), which is clinically more relevant than simple risk reclassification.
- Focus on randomised controlled trials and prospective observational cohort studies as the most reliable sources of evidence.
- Broad inclusion criteria to maximise generalisability of the findings.
- Key limitations reflect the scarcity of available evidence and include omission of potential harms of CACS screening, lack of economic assessments, insufficient data on clinical events and moderate to high risk of bias in most studies.
- Due to substantial heterogeneity among the included studies, a quantitative analysis was not possible.

## Introduction

 Cardiovascular disease (CVD) is the leading cause of death globally, claiming approximately 19.1 million lives annually.^1^ Notably, at least half of the fatalities and myocardial infarctions occur in subjects without a prior diagnosis of CVD.^2^ Even among those identified as ‘high-risk’ through risk stratification scores, the majority of cardiac events occur outside this group, highlighting the need for enhanced risk stratification methods.^3^ To improve risk prediction, incorporating risk modifiers such as non-invasive imaging of subclinical atherosclerosis has been proposed.^4 5^ Among these, coronary artery calcium scoring (CACS) has demonstrated superior performance in terms of discrimination and risk reclassification compared with other subclinical imaging markers (eg, ankle-brachial index or carotid ultrasound) or biomarkers in observational studies.^6 7^ The greatest clinical benefit of CACS may be in individuals classified at intermediate risk, and therefore guidelines recommend using CACS when an individual’s risk is near a decision threshold, such as determining the initiation of therapy in intermediate-risk individuals with high CACS.^4 5 8^ Also, many unblinded observational studies have shown good outcome prediction for CACS, with a CACS of 0 Agatston units (AU) being an outcome predictor for favourable outcomes.^9 10^

The increasing incorporation of CACS in clinical guidelines aligns with its broader adoption in clinical practice.^11^ However, accurate prediction alone does not necessarily lead to effective prevention.^12^ The clinical value of any marker must be evaluated based on its impact on patient management and outcomes.^13^ CACS can influence clinical practice and patient trajectories by prompting the initiation of preventive therapy (eg, lipid-lowering, blood-pressure lowering, antiplatelet) or providing intensive counselling regarding lifestyle, although no universally accepted score threshold has been identified. Conversely, potential harms of CACS include anxiety, subsequent invasive testing, increased healthcare utilisation, radiation exposure and false reassurance.^12 14^ To our knowledge, the last systematic review with a focus on CACS as a screening tool was published in 2014. Only 3 out of 15 studies were randomised controlled trials (RCTs), results could not be pooled and it concluded that CACS screening improved medication adherence with otherwise mixed results regarding other outcomes.^15^

This systematic review aimed to summarise the evidence from prospective studies on the impact of CACS for atherosclerosis screening on cardiovascular (CV) risk factor control, CV therapy, changes in health behaviour and clinical outcomes, compared with a control group not influenced by CACS results.

## Methods

The protocol for this review was registered with the PROSPERO International Prospective Register of Systematic Reviews. We used the Preferred Reporting Items for Systematic Reviews and Meta-Analyses checklist when writing our report.^16^

### Study selection

We included RCTs and prospective cohort studies with a control group, published between the inception of the database (Ovid MEDLINE, Embase and Cochrane Central Register of Controlled Trials) until 22 January 2025, without language restriction.

The studies included in this systematic review were required to meet the following criteria: (1) Adults without a history of, that is, asymptomatic for, clinical CVD, such as acute coronary syndrome (ACS), stroke, transient ischaemic attack (TIA) or peripheral arterial disease (PAD). (2) Imaging for CACS had to be performed using CT without contrast (ie, native). (3) In the intervention group, the CACS had to be communicated to the investigators, the participants or both, and/or be used for clinical decision-making. (4) Studies had to report follow-up clinical events or other pre-defined outcomes (see below). (5) The intervention group had to be compared with a control group of adults without CVD who either did not undergo CT for CACS or underwent CT for CACS but remained blinded to the results, along with the investigators.

The search strategy was not limited to RCTs due to the anticipated low number of such studies and the typically shorter follow-up periods associated with RCTs compared with observational studies. We excluded studies that solely assessed risk prediction, as this was not our primary research question. Additionally, the most recent systematic review on risk prediction has already recently been published, showing similar results to the 2018 United States Preventive Services Task Force (USPSTF) update.^17 18^

### Outcomes

The predefined outcomes were: (1) Clinical events including all-cause and CV mortality and non-fatal CV events (ACS, stroke, TIA, documented PAD), presented as overall incidence rate per 1000 person years, counts (n) and as incidence rate ratio for adverse events with a 95% CI. (2) Changes in CV risk factor control at follow-up (differences in smoking cessation rates, lipid level change, blood pressure (BP) change, diet control, weight reduction, differences in physical activity, blood sugar control), presented as median (25th, 75th percentile), n (%) or mean±SD change. (3) Differences in health behaviour (increased motivation to change lifestyle, enhanced perception of CV risk, adherence to medication, CV medication initiation), presented as a percentage difference.

### Search strategy

The first author developed a search strategy for each database with the assistance of an experienced medical information specialist. The detailed search strategies are presented in online supplemental file 1. Duplicate records were removed using Deduklick.^19^

### Data extraction and quality assessment

Two trained researchers (VS, LA) independently assessed titles and abstracts for eligibility. Articles identified by one or both researchers were included in a full-text review, which again was independently done independently by both researchers. Articles retained by both researchers after full-text screening were included in this review. Disagreements concerning eligibility in the full-text review were resolved by consensus, and involvement of a third researcher (MRB) in case of sustained disagreement. Both researchers extracted the data independently and in duplicate, and discussed and resolved disagreements by consensus or again through consultation of a third reviewer. Data extraction included characteristics of the screening intervention, type of study, baseline clinical characteristics of the participants and relevant outcomes. Both researchers independently assessed the quality of RCTs by the Cochrane risk-of-bias tool.^20^ For non-RCTs, the Newcastle-Ottawa scale was used.^21^ Disagreements were again resolved by consensus.

### Data analysis

We synthesised the systematic review qualitatively, as the data were too heterogeneous for quantitative synthesis with regard to population, interventions, outcomes. We summarised the characteristics and findings of the included studies in text and tables. We categorised our summaries of studies according to type of intervention, comparator, outcome and study design.

### Patient and public involvement

None.

## Results

**Figure 1:**
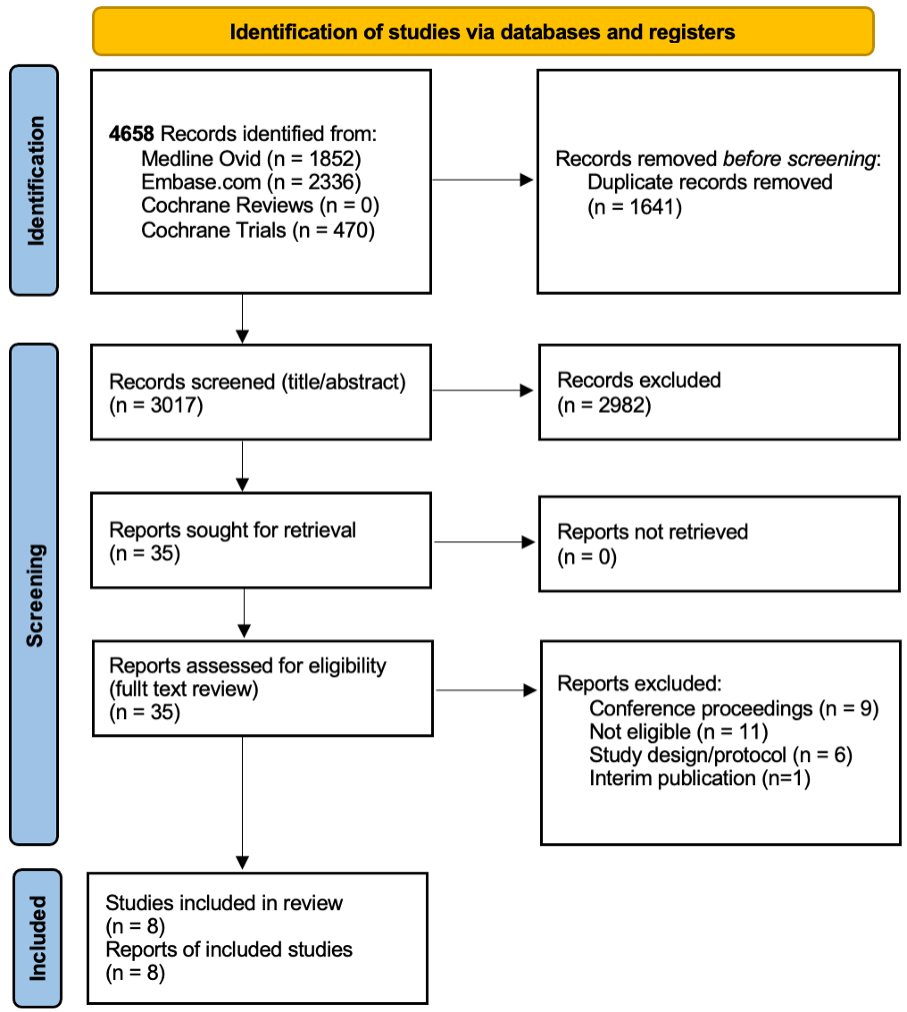
PRISMA 2020 flow diagram. PRISMA, Preferred Reporting Items for Systematic Reviews and Meta-Analyses.

After removing duplicates, a total of 3017 citations were identified using the described search algorithm. After a two-step screening process, seven RCTs and one observational study fulfilled the inclusion criteria (figure 1). The detailed characteristics of the studies are summarised in table 1. The studies were very diverse with regards to population and study design: the number of participants ranged from 56 to 43 447 with mean age ranging from 42 to 64 years, the proportion of women between 21% and 100% and follow-up duration ranging between 6 and 60 months. The distribution of baseline CV risk differed greatly across studies, with the RCT from Denissen *et al* including patients at increased risk (CACS group: ≥100 AU; Systematic Coronary Risk Evaluation Score (SCORE) group: >10%), and the RCT by O’Malley *et al* including a low-risk population with a mean 10-year Framingham Risk Score <6%, with approx. 20% having no modifiable risk factor and only 15% with detectable coronary calcifications in the CACS (CACS >0 AU).^22 23^ The interventions following CACS varied from communication of the results only to the participants, to initiation or recommendation of statin treatment after CACS screening depending on the study protocol. Treatment with a statin was either a fixed part of the intervention as with the study by Whitmore *et al* or recommended based on the CACS in the intervention arm or classical risk evaluations in the control group, as with the studies by Van Der Aalst *et al* and Muhlestein *et al*.^24^,^26^ The risk of bias of the studies generally was moderate to high (table 2).

**Table 1:** Study characteristics

Study	Intervention/control	Participants (%women)	Age (in years)	CACS (in AU)	Follow-up (in months)
Muhlestein *et al*(2022),^24^ RCT	Intervention: CACS and protocol-based statin recommendation communicated to participant and GPControl: PCE-score and protocol-based statin-recommendation communicated to participant and GP	n=601 (61%)	Range: 50–85Mean: CACS: 61Mean: Control: 61	0: 50%1–100: 36%>100: 14%	12
Whitmore *et al* (2024),^26^ RCT	Intervention: CACS, commencement of atorvastatin, if indicated antihypertensive therapy RF education and information of GPControl: Blinded CACS result, RF education and information of GP	n=449 (43%)	Range: 40–70Mean: 58Mean: CACS: 58Mean: Control: 58	>0: 100%Median: 33	36
van der Aalst *et al*(2020),^25^ RCT	Intervention: CACS and protocol-based recommendation of statins and ACEi to participants and GPControl: Screening by classical RF and guideline-based preventive treatment recommended to participant and GP	n=43.447 (42%)	Range: Men: 45–74Range: Women: 55–74Median: Women: 64 Median: Men: 59	>0:Women: 52%Men: 69%<100: 76%100–399: 15%>400: 8.9%	60
Denissen *et al*(2019),^22^ RCT	Same as van der Aalst *et al*^25^	n=600 (37%)	Range:Mean: 64Men: 45–74Women: 55–74	>100: 100%	15
Rozanski *et al*(2011),^27^ RCT	Intervention: CACS and RF counselling with recommendation to share CACS result with GPControl: RF counselling	n=2.137 (48%)	Range: 45–80Mean: 59	>0: 52%	48
Lederman *et al*(2006),^28^ RCT	Intervention: CACS and conventional screening with RF counselling and communicated to participant and GPControl: conventional screening with RF counselling	n=56 (100%)	Range: >55Mean: CACS: 64Mean: Control: 66	Mean 1.37	12
O'Malley *et al*(2003),^23^ 2×2 RCT	Intervention: CACS and disclosure of results to participant and GPControl: Blinded CACSSubgroups Intensive case management with guideline-based recommendation of treatment Usual care with guideline-based recommendation of treatment	n=450 (21%)	Range: 39–45Mean: 42	>0: 15%	12
Chi *et al*(2014),^29^ Observational study	Intervention: Patients who received a CACS ordered by their physicianControl: Patients who were denied a CACS ordered by their physician	n=3.814 (41%)	Range: 18–64Mean: 53	No information	6 for therapeutic interventions23 for clinical events

ACEi, ACE inhibitor; AU, Agatston units; CACS, coronary artery calcium score; GP, general practitioner; PCE, pooled cohort equation; RCT, randomised controlled trial; RF, risk factor.

**Table 2:** Risk of bias assessment done by the revised Cochrane risk-of-bias tool for randomised trials (RoB 2) and with the Newcastle-Ottawa scale for non-RCTs

Study	Randomisation process	Deviations from the intended interventions		Missing outcome data	Measurement of the outcome	Selection of the reported result	Overall
		Effect of assignment to intervention	Effect of adhering to intervention				
Muhlestein *et al*(2022),^24^ RCT	Low	Low	Some concerns	High	Some concerns	Low	High
Whitmore *et al* (2024),^26^ RCT	Low	Some concerns	Some concerns	Low	High	Low	High
van der Aalst *et al*(2020),^25^ RCT	Low	Low	Low	Low	Low	Low	Low
Denissen *et al*(2019),^22^ RCT	Low	Low	Low	Low	Some concerns	Low	Some concerns
Rozanski et al(2011),^27^ RCT	Some concerns	Low	Low	High	Some concerns	Low	High
Lederman *et al*(2006),^28^ RCT	Some concerns	Low	High	High	Some concerns	Some concerns	High
O'Malley *et al*(2003),^23^ 2×2 RCT	Low	Low	Some concerns	Low	Low	Low	Some concerns

Study	Selection	Comparability	Outcome	Overall
Chi *et al*(2014),^29^ Observational study	3/4	0/1	1/3	4/8

RCT, randomised controlled trial.

### Effects on CV risk factor control

The effects of CACS screening on the outcomes are summarised in online supplemental table 1, and presented in detail in online supplemental tables 2–9. Five RCTs evaluated at least one CV risk factor as an outcome. Three of the trials (Muhlestein *et al*, Whitmore *et al* and Rozanski *et al*) showed a favourable improvement,^24 26 27^ one trial an unfavourable deterioration^28^ and one trial found no significant difference in CV risk factor control.^23^

Of the three trials demonstrating an improvement, all showed a benefit in blood lipid control (absolute between-group change of LDL cholesterol −6.0 to −44.9 mg/dL).^24 26 27^ Only Rozanski *et al* demonstrated a statistically significant improvement in systolic BP between intervention and control groups (n=2137; baseline: CACS: 130 mm Hg (119, 142), control: 131 mm Hg (121, 144), p=0.03; change: CACS: −5 mm Hg (−16 to −6), control: −7 mm Hg (−18 to −3), p=0.02), as well as a benefit on waist circumference (CACS: change: 0 in (−3, 2); control: change: 1 in (−2, 3); p=0.01).^27^ The trial by Lederman *et al* (n=56) was the only trial showing a deterioration in control of a CV risk factor. In its CACS-guided group, participants had a lower decrease in total cholesterol and an increase in LDL cholesterol levels (CACS: change: +0.03±0.69 mmol/L; control: change: −0.45±0.75 mmol/L).^28^

### Effects on CV preventive medication and adherence

Six of the seven included RCTs and the observational study described an effect of CACS on the use of CV medication, with diverging results. Muhlestein *et al* and van der Aalst *et al* showed a decrease in protocol-based indicated CV medication in the CACS group compared with a group who was screened by classical risk factors. Denissen *et al*, who compared an increased risk CACS group (CACS >100 AU) to an increased risk control group (SCORE moderate or high), found an increase in protocol-based indicated CV medication in the CACS group.^22 24 25^ Rozanski *et al* showed an increase in newly prescribed medication after 4 years in the CACS group compared with the control group.^27^ In the trial by Whitmore *et al*, there was a difference in statin utilisation at 36 months (CACS: 89%; intervention: 13%), as the intervention included initiating statin therapy after CACS, regardless of the results. However, no differences were observed in BP or diabetes medication at follow-up.^26^ Lederman *et al* and Chia-hsuan Chi *et al* found no change in the usage of CV medication during follow-up in the CACS group compared with the control group.^28 29^

Of the trials showing a decrease, van der Aalst *et al* reported a decrease in indicated CV preventive medication overall after screening with CACS (men: CACS: 30.7%, control: 43.2%, p<0.001; women: CACS: 16.8%, control: 26.7%, p<0.001).^25^ Significantly fewer participants in the trial of Muhlestein *et al* had an indication for a statin in the CACS group (CACS: 35.9%; control: 47.9%; p=0.005).^24^

Two trials showed an increase in the usage of CV medications.^22 27^ Denissen *et al* reported an increase in indicated CV medication as well as initiated overall CV medication (CACS: 46.4%; control 20%; p<0.001) with a significant increase in CVD medication for antihypertensive and lipid-lowering medication in the CACS group.^22^ Rozanski *et al* did not investigate the difference in indicated CV medication, as their study protocol consisted only of a CV risk factor education without treatment recommendations, but they showed an increase in new antihypertensive medication (CACS: 24%; control: 18%; p=0.02) and a trend towards an increase in lipid-lowering medication after 4 years of follow-up in the CACS group.^27^

Lederman *et al* and Chia-hsuan Chi *et al* only reported the usage of CV medication at follow-up and showed no difference in antihypertensive or lipid-lowering medication or any other investigated medication in the CACS group compared with the control group.^28 29^

The adherence to medication was investigated in three trials. Muhlestein *et al* observed a significant difference in adherence for statins, favouring the intervention group (adherence at 12 month follow-up, CACS: 63.3%; control: 45.6%; p=0.03).^24^ In the trials by Denissen *et al* and Rozanski *et al*, the adherence in both groups was high independently of CACS.^22 27^

### Effects on behaviour

Five RCTs assessed behavioural changes of the participants. Denissen *et al* showed that a significantly higher proportion in the intervention group consulted their general practitioner (GP) after the intervention (CACS: 94%; control: 62.8%; p=0.002) with the most common stated reason being the desire to lower CV risk.^22^ All other trials examined behavioural variables such as dietary changes (fibre, fat), smoking, physical activity, and various well-being scores (‘heart healthy and sustained’, ‘perceived wellness’, ‘objective wellness’, ‘depression score’, ‘anxiety’, ‘stress’, ‘mental health and functional status’ and ‘motivation to change lifestyle’). Among these, only Whitmore *et al* reported a significantly higher percentage of self-reported adherence to daily exercise (CACS: 108 (96%); control: 68 (59%); p<0.001) at 36 months. All other trials found no significant between-group differences in any of the aforementioned behavioural variables.^2326^,^28^

The trial by Muhlestein *et al* investigated the response rate of GPs to recommendations, which showed a higher communication of study recommendations in the intervention group (overall response rate CACS: 47.3% vs control: 39.0%; p=0.05).^24^

### Clinical events

Two RCTs and the observational study (combined n=6552), reported a total of 52 clinical events, leading to a calculated overall incidence rate of approximately 3.16 events per 1000 person-years across all. The number of events in the two RCTs was so low (n=34) that they deemed a quantitative analysis inadequate due to insufficient power.^24 27^ Chia-hsuan Chi *et al* analysed their clinical events, which were similarly low (n=18; composite of acute myocardial infarction, stroke and unstable angina pectoris) and concluded that there was no significant between-group difference with an age-sex adjusted incidence rate ratio for adverse events of +1.1 (95% CI 0.36 to 3.38).^29^

## Discussion

In this systematic review of seven RCTs and one prospective cohort study, with a total of 51 554 participants, investigating the impact of screening with CACS in adults in a primary prevention setting with asymptomatic participants, we found generally favourable effects on CV risk factor control, diverging results on indicated or used CV medication, a possibly favourable effect on medication adherence, motivation to change and physical activity. Data were insufficient for an adequate analysis of the effect on clinical endpoints.

The studies in this review that demonstrated a favourable effect on CV risk factors generally had a higher burden of CACS and/or CV risk factors compared with those showing no effect or an unfavourable effect. This suggests that improvements in CV risk factor control may be primarily driven by the presence of CACS and/or CV risk factors, as the presence of disease likely serves as a motivating factor.^23^ Lederman *et al* proposed that a low prevalence of CV risk factors might even lead to an unfavourable effect.^28^ However, the trial by O’Malley *et al*, which had a similarly low prevalence of CV risk factors, showed no such effect, similar to another RCT of carotid ultrasound with no impact on smoking cessation and control of CV risk factors.^30^ It is important to note that the study by Lederman *et al* had several limitations, including a high risk of bias.

The utilisation and adherence to CV medication could explain the positive effect on CV risk factor control observed in some studies. Differences in study results likely stem from variations in population characteristics and intervention protocols. Studies involving intermediate-risk populations and those with protocol-based treatment recommendations demonstrated a significant decrease in indicated CV medication use.^24 25^ However, the CACS-guided groups showed higher medication adherence, leading to more effective reductions in LDL cholesterol. In the trial by Denissen *et al*, the study protocol recommended starting a statin and an ACE inhibitor in all participants in the increased CACS group, while drug treatment in the control group was only recommended when the systolic BP exceeded 140 mm Hg and/or LDL cholesterol exceeded 5 mmol/L. This resulted in more indicated CV medication in the CACS group; and additionally, the percentage of actually prescribed CV medication by GPs was significantly higher in this group. The difference in statin utilisation in the trial by Whitmore *et al* was due to the intervention, which included starting a statin for all participants in the intervention group. In contrast, GPs of control group participants were only informed of their intermediate risk without a treatment recommendation. This led to a significant difference in statin use at 36 months, explaining the greater reduction in blood lipids, which is likely attributable more to the co-intervention of statin initiation than to CACS itself.^26^ Conversely, Rozanski *et al*, who did not implement a protocol-based treatment recommendation but only counselled participants on their risk factors, observed an increase in antihypertensive and lipid-lowering medication at 4 years of follow-up. These findings suggest that, depending on the protocol-based algorithm, newly indicated CV medication might decrease as CACS reclassifies more patients to a lower risk. However, in most studies, medication adherence by participants and/or adherence of the GP to treatment recommendations was higher in the CACS group. This increased adherence may be due to both patients and GPs prioritising the results of CACS over conventional CV risk factor screening. Supporting this, the trial by Denissen *et al* observed that participants in the CACS group consulted their GPs more frequently and more promptly.^22^ However, this does not seem to consistently translate into changes in health behaviour during follow-up, as most studies found no significant differences in other behavioural aspects examined.

Our review found insufficient data to assess whether these favourable effects of screening with CACS in asymptomatic patients have a beneficial effect on clinical endpoints such as CV events or mortality, or whether reclassification with fewer indicated CV medication might even have an unfavourable effect on these endpoints.

The strengths of this review are the focus on RCTs and prospective observational cohort studies, as these are the most reliable sources of evidence. Also, this presents the first systematic review focusing on the screening with CACS, which is clinically more relevant than simple risk reclassification. One limitation of this systematic review is that we did not consider the potentially harmful aspects of screening with CACS, as recent and thorough reviews on this subject are already available.^18^ Potentially harmful effects include radiation, anxiety, downstream testing (follow-up CTs, biopsies, bronchoscopies, etc) and financial burden. The studies included in this review showed no differences between groups regarding mental health outcomes. Nevertheless, these harmful aspects must be considered when implementing a screening tool, especially in the absence of strong evidence on its benefits. Additional limitations of our review include the heterogeneity of the included studies, which prevented pooling of the results, and the lack of sufficient data on clinical events. The heterogeneity also arises from differences in publication dates, the adaptation of guidelines over time, and variations between guidelines in different countries. Finally, we did not assess the economic aspects of screening with CACS.

Rodondi *et al* conducted a systematic review on atherosclerosis screening using various non-invasive imaging techniques and found limited evidence, advocating for large clinical trials before widespread adoption. Since then, CACS has emerged as the preferred imaging method recommended in most guidelines, prompting the focus of this review. Some large-scale RCTs have since been published and are discussed herein, with more underway. The ‘CACS Women’s Trial’ for example, currently in the recruiting phase, targets women with low or intermediate risk and at least one novel CVD risk factor—a traditionally overlooked population.^31^ Despite the challenges of conducting trials powered for clinical events, which have previously hindered promising trial designs like the VIEW trial due to funding issues, progress is being made.^32^ Notable examples include the CorCal and ROBINSCA trials, with preliminary results presented in this review and full clinical event data anticipated soon.^24 25^ A significant RCT garnering attention is the DANCAVAS trial, which investigates the impact of a comprehensive screening process, including CACS.^33^ While their preliminary 5-year outcomes did not show a reduction in all-cause mortality, there was a pattern towards favourable effects in younger men.^34^ However, because the DANCAVAS trial did not restrict inclusion to participants without CVD, it was ineligible for this review as it does not strictly address a primary prevention setting. The SCOT-Heart 2 trial will address the effect of screening with CT angiography, which adds more information due to the use of contrast, but on the other hand makes the intervention more demanding and should be the subject of a different review.^35^

In conclusion, screening with CACS compared with conventional screening methods in intermediate to high-risk populations with a CACS-guided intervention might have favourable effects on CV risk factor control and on CV medication adherence in asymptomatic adults in primary prevention settings. However, its impact on health-related behaviours remains inconsistent. Whether these putative benefits translate into improved clinical outcomes, such as reduced CV events or mortality, currently remains unclear. Until ongoing large-scale trials provide definite evidence on whether enhanced prediction leads to better prevention, despite its proven clinical benefit in predicting outcomes in individual patients, the clinical use of CACS for screening purposes in asymptomatic patients remains uncertain.

## Supplementary material

10.1136/bmjopen-2025-101472 online supplemental file 1

## Data Availability

No data are available.
