# Supplementary material for: Coronary atherosclerosis screening in asymptomatic adults using coronary artery calcium for cardiovascular prevention: a systematic review of randomised controlled trials and prospective cohorts
Source: BMJ Open. 2025 Jul 5;15(7):e101472. doi: 10.1136/bmjopen-2025-101472 (PMC12228433; doi:10.1136/bmjopen-2025-101472)
Supplement: online supplemental file 1 [file bmjopen-15-7-s001.docx]

Appendix

**Full Search Strategy per Database**

Requester: Victor Scheu, Supervisor: Manuel Blum

Research question:
**Atherosclerosis screening in asymptomatic adults by CAC for cardiovascular prevention: systematic review**

**Overview databases & results**

Date of 1^st^ search: **Nov 29, 2022** Date last searched: **Jan 22, 2025**

|  | Before deduplication | After deduplication |  | Before deduplication | After deduplication |
| --- | --- | --- | --- | --- | --- |
| Medline ALL Ovid | 1613 | 1611 |  | 1852 | 1851 |
| Embase.com | 2011 | 784 |  | 2336 | 942 |
| Cochrane Reviews  Cochrane Trials | 13  425 | 13  208 |  | 0  470 | 0  224 |
| Total | 4'062 | **2’616** |  | 4’658 | **3’017** |

1446 duplicate records removed using ^1^Deduklick 1641 duplicates removed using ^1^Deduklick

^[1]^. Borissov, N.; Haas, Q.; Minder, B.; Kopp-Heim, D.; von Gernler, M.; Janka, H.; Teodoro, D.; Amini, P. Reducing systematic review burden using Deduklick: A novel, automated, reliable, and explainable deduplication algorithm to foster medical research. Syst. Rev. 2022, 11, 172. https://doi.org/10.1186/s13643-022-02045-9

**Search concepts** (block-building approach according to PICO)

**Embase.com**
Combination of search concepts: 1) AND 2) AND 3) NOT 4)

| **1) Coronary Artery Calcification (CAC) imaging, done by a CT: CAC-CT** |
| --- |
| ('coronary artery calcium score'/exp OR 'coronary artery calcium'/de OR ('coronary artery calcification'/de AND 'computer assisted tomography'/de) OR ((("coronary artery calcium" OR "coronary artery calcificat*" OR "coronary calcium" OR CAC OR "coronary calcificat*") NEAR/5 (scor* OR scan* OR screening OR testing* OR check* OR assess* OR imaging* OR trial* OR "computed tomograph*" OR "electron beam tomograph*" OR "electron beam CT" OR EBCT OR "cardiac-CT" OR CT)) OR CAC-CT OR "total-body-screening*" OR (calcium NEXT/2 scor*) OR Agatston):ab,ti) |
| **2) Asymptomatic population: without clinical cardiovascular disease (CVD)** |
| ('asymptomatic disease'/exp OR 'screening'/de OR 'screening test'/de OR 'primary prevention'/de OR 'prophylaxis'/de OR (asymptomatic OR inapparent* OR non-symptomatic OR nonsymptomatic OR pre-symptomatic OR presymptomatic OR pre-clinical OR preclinical OR sub-clinical OR subclinical OR symptom-less OR symptomless OR "general population" OR normal OR non-high-risk OR "free of" OR symptomfree OR without OR healthy OR pre-diabet* OR prediabet* OR screening* OR "primary prevention" OR "primary prophylaxis"):ti,ab) |
| **3) Study design:  RCTs** (based on Cochrane highly sensitive search strategy for identifying RCTs: Sensitivity-maximizing version (2008) **Observational studies, only if** containing a **control group**  Search strategies for observational study design filters are based on: Francis A. Countway Library of Medicine, Harvard University; <https://guides.library.harvard.edu/meta-analysis> Methodology Filters: Observational Designs |
| ('randomized controlled trial'/exp OR 'controlled clinical trial'/exp OR randomized:ti,ab OR placebo:ti,ab OR 'drug therapy':lnk OR randomly:ti,ab OR trial:ti,ab OR groups:ti,ab OR blind*:ti,ab  **OR**  ('controlled study'/exp OR 'major clinical study'/exp OR 'prospective study'/exp OR 'cohort analysis'/exp OR 'cohort':ti,ab OR 'compared':ti,ab OR 'groups':ti,ab OR follow-up*:ti,ab OR followup*:ti,ab OR followed-up:ti,ab OR ((prospectiv* OR populat* OR observat* OR intervent* OR clinical OR comparative OR longitudinal) NEXT/1 (stud* OR trial*)):ti,ab  **AND**  (group OR groups OR arm OR "reference patient*" OR ((subject* OR patient*) NEAR/3 random*) OR "healthy control*" OR "population control*" OR controls):ti,ab)) |
| **4) Limits:  - excl. animal studies, study types like editorials, comments etc., excl. case reports, retrospective studies - filter for adults (excl. studies with children or adolescents)** |
| NOT ([animals]/lim NOT [humans]/lim) NOT ([Conference Abstract]/lim OR [Letter]/lim OR [Note]/lim OR [Editorial]/lim OR 'retrospective study'/de OR 'case report'/de) NOT (([infant]/lim OR [child]/lim OR [adolescent]/lim) NOT [adult]/lim) |

**eAppendix - Documentation of all search strategies used for this review**

**Embase.com** (via <https://www.embase.com>)

('coronary artery calcium score'/exp OR 'coronary artery calcium'/de OR ('coronary artery calcification'/de AND 'computer assisted tomography'/de) OR ((("coronary artery calcium" OR "coronary artery calcificat*" OR "coronary calcium" OR CAC OR "coronary calcificat*") NEAR/5 (scor* OR scan* OR screening OR testing* OR check* OR assess* OR imaging* OR trial* OR "computed tomograph*" OR "electron beam tomograph*" OR "electron beam CT" OR EBCT OR "cardiac-CT" OR CT)) OR CAC-CT OR "total-body-screening*" OR (calcium NEXT/2 scor*) OR Agatston):ab,ti) **AND** ('asymptomatic disease'/exp OR 'screening'/de OR 'screening test'/de OR 'primary prevention'/de OR 'prophylaxis'/de OR (asymptomatic OR inapparent* OR non-symptomatic OR nonsymptomatic OR pre-symptomatic OR presymptomatic OR pre-clinical OR preclinical OR sub-clinical OR subclinical OR symptom-less OR symptomless OR "general population" OR normal OR non-high-risk OR "free of" OR symptomfree OR without OR healthy OR pre-diabet* OR prediabet* OR screening* OR "primary prevention" OR "primary prophylaxis"):ti,ab) **AND** ('randomized controlled trial'/exp OR 'controlled clinical trial'/exp OR randomized:ti,ab OR placebo:ti,ab OR 'drug therapy':lnk OR randomly:ti,ab OR trial:ti,ab OR groups:ti,ab OR blind*:ti,ab **OR** ('controlled study'/exp OR 'major clinical study'/exp OR 'prospective study'/exp OR 'cohort analysis'/exp OR 'cohort':ti,ab OR 'compared':ti,ab OR 'groups':ti,ab OR follow-up*:ti,ab OR followup*:ti,ab OR followed-up:ti,ab OR ((prospectiv* OR populat* OR observat* OR intervent* OR clinical OR comparative OR longitudinal) NEXT/1 (stud* OR trial*)):ti,ab **AND** (group OR groups OR arm OR "reference patient*" OR ((subject* OR patient*) NEAR/3 random*) OR "healthy control*" OR "population control*" OR controls):ti,ab)) **NOT** ([animals]/lim **NOT** [humans]/lim) **NOT** ([Conference Abstract]/lim OR [Letter]/lim OR [Note]/lim OR [Editorial]/lim OR 'retrospective study'/de OR 'case report'/de) **NOT** (([infant]/lim OR [child]/lim OR [adolescent]/lim) **NOT** [adult]/lim)

**Medline ALL** (via Ovid)

((Coronary Vessels/ AND Calcium/) OR (Coronary Vessels/ AND Calcinosis/) OR exp Vascular Calcification/dg OR ((("coronary artery calcium" OR "coronary artery calcificat*" OR "coronary calcium" OR CAC OR "coronary calcificat*") ADJ5 (scor* OR scan* OR screening OR testing* OR check* OR assess* OR imaging* OR trial* OR "computed tomograph*" OR "electron beam computed tomograph*" OR "electron beam CT" OR EBCT OR "cardiac-CT" OR CT)) OR CAC-CT OR "total-body-screening*" OR (calcium ADJ2 scor*) OR Agatston).ab,ti.) **AND** (asymptomatic diseases/ OR mass screening/ OR primary prevention/ OR (asymptomatic OR inapparent* OR non-symptomatic OR nonsymptomatic OR pre-symptomatic OR presymptomatic OR pre-clinical OR preclinical OR sub-clinical OR subclinical OR symptom-less OR symptomless OR "general population" OR normal OR non-high-risk OR "free of" OR symptomfree OR without OR healthy OR pre-diabet* OR prediabet* OR screening* OR "primary prevention" OR "primary prophylaxis").ab,ti.) **AND** (randomized controlled trial/ OR controlled clinical trial/ OR clinical trials as topic/ OR clinical trial/ OR randomized.ab. OR placebo.ab. OR drug therapy.fs. OR randomly.ab. OR trial.ab. OR groups.ab. OR blind*.ab. **OR** (cohort studies/ OR follow-up studies/ OR longitudinal studies/ OR prospective studies/ OR comparative study/ OR (cohort OR compared OR groups OR follow-up* OR followup* OR followed-up OR ((prospectiv* OR populat* OR observat* OR intervent* OR clinical OR comparative OR longitudinal) ADJ (stud* OR trial*))).ab,ti. **AND** (group OR groups OR arm OR "reference patient*" OR ((subject* OR patient*) ADJ3 random*) OR "healthy control*" OR "population control*" OR controls).ab,ti.)) **NOT** (exp animals/ NOT humans/) **NOT** (letter OR news OR comment OR editorial OR congress).pt. **NOT** (retrospective studies/ OR case reports/) **NOT** ((exp infant/ OR exp child/ OR adolescent/) NOT (exp adult/ OR female/ OR male/))

**Cochrane Database of Systematic Reviews (via Wiley)**Issue 11 of 12, November 2022 / update search: Issue 1 of 12, January 2025
**Cochrane Central Register of Controlled Trials (via Wiley)**Issue 11 of 12, November 2022 / update search: Issue 12 of 12, December 2024
(Word variations have been searched)

((((coronary-artery-calcium OR coronary-artery-calcificat* OR coronary-calcium OR CAC OR coronary-calcificat*) NEAR/5 (scor* OR scan* OR screening OR testing* OR check* OR assess* OR imaging* OR trial* OR tomograph* OR electron-beam-CT OR EBCT OR cardiac-CT OR CT)) OR CAC-CT OR total-body-screening* OR (calcium NEXT/2 scor*) OR Agatston):ab,ti) **AND** ((asymptomatic OR inapparent* OR non-symptomatic OR nonsymptomatic OR pre-symptomatic OR presymptomatic OR pre-clinical OR preclinical OR sub-clinical OR subclinical OR symptom-less OR symptomless OR "general population" OR normal OR non-high-risk OR "free of" OR symptomfree OR without OR healthy OR pre-diabet* OR prediabet* OR screening* OR "primary prevention" OR "primary prophylaxis"):ti,ab)

Tabelle 1 Effect of CACS on outcomes

|  | Change/Utilization of CV medication | | | | Adherence to medication | | | | Health behavior changes | | | | | CV risk factor control | | | | Clinical events |
| --- | --- | --- | --- | --- | --- | --- | --- | --- | --- | --- | --- | --- | --- | --- | --- | --- | --- | --- |
|  | **Overall** | **Blood pressure** | **Lipid** | **Diabetic** | **Overall** | **Blood pressure** | **Lipid** | **Diabetic** | **Smoking** | **Diet** | **Physical activity** | **«Well-being»- Scores** | **Motivation to change** | **Blood pressure** | **Blood lipids** | **Weight**  ****** | **Glucose/**  **HbA1c** |  |
| Muhlestein  et al. |  |  | **-** |  |  |  | ↑ |  |  |  |  |  |  |  | ↑ |  |  | * |
| Whitmore et al. |  | ↔ | **+** | ↔ |  |  |  |  | ↔ | ↔ | ↑ | ↔ |  | ↔ | ↑ | ↔ | ↔ |  |
| van der Aalst et al. | **-** |  |  |  |  |  |  |  |  |  |  |  |  |  |  |  |  |  |
| Denissen  et al. | **+** | **+** | **+** |  | ↔ |  |  |  |  |  |  |  | ↑ |  |  |  |  |  |
| Rozanski  et al. |  | **+** | ↔ | ↔ |  | ↔ | ↔ | ↔ | ↔ |  | ↔ |  |  | ↑ | ↑ | ↑ | ↔ | * |
| Lederman  et al. |  | ↔ | ↔ |  |  |  |  |  |  | ↔ |  | ↔ |  | ↔ | ↓ | ↔ | ↔ |  |
| O'Malley  et al. |  |  |  |  |  |  |  |  | ↔ |  | ↔ | ↔ | ↔ | ↔ | ↔ | ↔ | ↔ |  |
| Chia-hsuan Chi et al. |  | ↔ | ↔ |  |  |  |  |  |  |  |  |  |  |  |  |  |  | ↔ |

↑ Improvement or better controlled; ↓ less controlled; + increase; - decrease; ↔ no significant effect; * statistical power was insufficient for an adequate assessment; **weight-criterion includes BMI and/or weight and/or waist circumference; CV: cardiovascular

Table 2 Investigated behavioral variables part 1

|  | **O'Malley et al.** | **Rozanski et. al.** | **Venkataraman et.al.** | **Denissen et al.** | **Whitmore et al.** |
| --- | --- | --- | --- | --- | --- |
| Smoking, n (%) | CACS: Baseline: 18 +-7.8; change: 5/13  Control: Baseline: 20 +-9.2; change: 4/17  p=0.63 |  | CACS: Baseline: 10/221 (4.5%); at 12 months: 2%  Control: Baseline: 9/222 (4.1%); at 12 months: 3%  OR 0.88 (0.35, 2.18; p=0.78) |  | CACS: Baseline: 4.5%,  at 36 months: 2.5%;  Control: Baseline: 4.4%  At 36 months: 3.8%  Difference: -1.6% (-5.0 to 1.9) |
| Quit smoking, n (%) |  | CACS: 34/69 (49%)  Control: 16/36 (44%)  p=0.64 |  |  |  |
| Physical activity^[[1]](#footnote-1)^ | CACS: Baseline: 3.0 +-0.85; change: 0.02 +-0.05;  Control: Baseline: 2.9 +-0.87; change: -0.08 +-0.05  p=0.23 |  | CACS: Baseline: ?;  at 12 months: 87%;  Control: Baseline: ?;  at 12 months: 88%  OR 0.89 (0.4, 1.61)  p=0.80 |  | Adherance to daily exercise  CACS: n=108 (95.6%)  Control: n=68 (59.1%)  p< 0.001 |
| Exercise  (≥ 3 times per week), n (%) |  | CACS: 214/582 (37%)  Control: 95/266 (36%); p=0.77 |  |  |  |
| GP consult^[[2]](#footnote-2)^/  motivation to change^[[3]](#footnote-3)^, n (%) | CACS: Baseline: 8.3 +-1.3; change: 0.27 +-0.0.9  Control: Baseline: 8.3 +- 1.5; change: 0.38 +-0.12  p=0.48 |  |  | CACS: 140/149 **(94%)**  Control: 86/137 (62.8%)  p=0.002 |  |

Table 3 Investigated behavioral variables part 2

|  | **O'Malley et al.** | **Whitmore et al.** | **Lederman et al.** |
| --- | --- | --- | --- |
| Depression score | PRIME-MD score  CACS: -0.04 +-0.21  Control: -0.13 +-0.22  p=0.75 | PHQ-9  CACS: change at 36 months: 0.1 (-0.2 to 0.5)  Control: change at 36 months -0.1 (-0.5 to 0.2)  Difference in difference (DID):  0.3 (-0.2 to 0.7) p=0.27 |  |
| Anxiety score | PRIME-MD score  CACS: -0.19 +-0.18  Control: -0.38 +-0.21  p=0.50 | GAD-7  CACS: change at 36 months: 0.06 (-0.3 to 0.5)  Control: change at 36 months -0.1 (-0.4 to 0.1)  DID: 0.2 (-0.2 to 0.7); p=0.35 |  |
| Stress score^[[4]](#footnote-4)^ | CACS: -0.51 +-0.19  Control: -0.62 +-0.17  p=0.67 |  |  |
| Mental health functional status | Short Form-36 score  CACS: 0.44 +-0.55  Control: 1.01 +- 0.48  p=0.44 |  |  |
| Health status  EQ5D-5L VAS |  | CACS: change at 36 months: -0.8 (-2.5 to 0.8)  Control: change at 36 months 0.1 (-0.5 to 2.4)  DID: -1.9 (-4.1 to 0.2): p=0.08 |  |
| Fiber intake, n of patients with change of intake |  |  | CACS: change: 15 (65.2%)  Control: change: 16 (57.1%)  p=0.3782 |
| Fat intake, n of patients with change of intake |  |  | CACS: change: 16 (66.7%)  Control: change: 22 (78.6%)  p=0.6130 |

Table 4 Two RCTs and one observational study (Chia et al) investigating clinical events

|  | **Rozanski et. Al.** | **Chia-hsuan Chi et.al.** | **Muhlestein JB et.al.** |
| --- | --- | --- | --- |
| Acute stroke, n |  | CACS: 4 +-0.24  Control: 0 +-0.00  p=0.58 | CACS: 1 (before recommendation)  Control: 0 |
| Unstable AP, n |  | CACS: 4 +-0.24  Control: 3 +-0.56  p=0.42 | CACS: 1  Control: 0 |
| Combined number of deaths and/or myocardial infarction, n | CACS: 27 (2.1%)  Control: 6 (1.0%)  p=0.08 |  |  |
| All-cause death, n | CACS: 17 (1.3%)  Control: 4 (0.6%)  p=0.24 |  |  |
| Cardiac death, n | CAC: 2 (0.2%)  Control: 1 (0.2%)  p=1.00 |  |  |
| Acute MI, n | CAC: 10 (0.8%)  Control: 2 (0.3%)  p=0.36 | CACS: 8 +-0.49  Control: 2 +-0.37  p=0.73 |  |
| Composite Acute MI, stroke, unstable AP, n |  | CACS: 14 +-0.85  Control: 4 +-0.74; p=0.79  The age-sex adjusted incidence rate ratio for adverse events was 1.1 (95% CI, 0.36-3.38) |  |

Table 5 Investigated differences in medical treatment part 1

|  | **Alan Rozanski et. Al.** | **Sabine JAM Denissen et.al.** | **Muhlestein JB et.al.** |
| --- | --- | --- | --- |
| **New medication overall**, n (%) |  | CACS: 65/140 (46.4%); Control: 16/80 (20%); p<0.001 |  |
| New lipid meds, n (%) | CACS: 284/963 (29%); Control: 109/441 (25%); p=0.06 |  |  |
| **New BP meds**, n (%) | CACS: 214/877 (24%); Control: 77/419 (18%); p=0.02 |  |  |
| New diabetic meds, n (%) | CACS: 40/1260 (3%); Control: 15/595 (3%); p=0.44 |  |  |
| New Aspirin, n (%) | CACS: 92/1130 (8%); Control: 39/525 (7%); p=0.62 |  |  |
| Adherance to medication overall, n (%) |  | CACS: 104/106 (98.1%); Control: 35/35 (100%) |  |
| Adherance to lipid meds, n (%) | CACS: 281/325 (86%); Control: 145/168 (86%); p=0.96 |  |  |
| Adherance BP meds, n (%) | CACS: 388/414 (94%); Control: 173/192 (90%); p=0.11 |  |  |
| Adherance diabetic meds, n (%) | CACS: 45/51 (88%); Control: 26/28 (93%); p=0.71 |  |  |
| Adherance Aspirin, n (%) | CACS; 43/158 (27%); Control: 26/84 (31%); p=0.54 |  |  |
| **Adherance to statins**, % |  |  | CACS 63.3%; Control: 45.6%; (p = 0.03) |
| **Utilization of any CV medication after intervention overall**, n (%) |  | CACS 108/140 (77.1%); Control: 35/80 (12.5%); p<0.001 |  |
| **Statin recommendation**, % |  |  | CACS: 35.9%; Control: 47.9%; (p = 0.005) |

Table 6 Investigated differences in medical treatment part 2

|  | **Chia-hsuan Chi et.al.** | **Denissen et.al.** |
| --- | --- | --- |
| Utilization of ACEi or ARB after intervention, n | CACS: 186 +-17.45;  Control: 122 +-18.40; p=0.61 |  |
| Utilization of Beta-blockers after intervention, n | CACS: 4 +-0.38;  Control: 4 +-0.60; p=0.50 |  |
| Utilization of Calcium channel blockers after intervention, n | CACS: 54 +-5.07;  Control: 38 +-5.73; p=0.55 |  |
| Utilization of Diuretics after intervention, n | CACS: 9 +-0.84;  Control: 9 +-1.36; p=0.31 |  |
| Utilization of Nitrates after intervention, n | CACS: 16 +-1.50;  Control: 4 +-0.60; p=0.11 |  |
| Utilization of Statins after intervention, n | CACS: 379 +-35.55;  Control: 233 +-35.14; p=0.86 |  |
| Utilization of BP lowering after intervention, n |  | **CACS**: 82/140 **(58.6%);**  **Control** 27/80 **(33.8%);** p<0.001 |
| Utilization of Cholesterol lowering after intervention, n (%) |  | **CACS**: 93/140 **(66.4%);**  **Control** 18/80 **(22.5%);** p<0.001 |
| Utilization of Cholesterol + BP lowering after intervention, n(%) |  | **CACS:** 67/140 **(47.9%);**  **Control** 10/80 **(12.5%);** p<0.001 |

Table 7 Investigated differences in medical treatment part 3

|  | **Whitmore et al.** | **Van der Aalst et. Al** | **Lederman et al.** |
| --- | --- | --- | --- |
| **New medication indicated/recommended after screening**, n (%) |  | Men:  **CACS:** 2068/6727 **(30.7%);**  **Control:** 2666/6176 **(43.2%)**  (p<0.001; absolute reduction of 12.4%);  Women:  CACS: 1043/6223 (16.8%);  Control: 1604/6009 (26.7%);  p<0.001; absolute reduction of 9.9% |  |
| BP meds increase or new, n (%) | CACS: Change at 3y: 6.6%  Controle: Change at 3y: 9.9%  DID: -3.6 (-10.6 to 3.4) |  | CACS: change: 2 (7.7%);  Control: change: 4 (13.3%); p=0.6749 |
| Lipid meds increase or new, (%) |  |  | CACS: change: 2 (7.7%);  Control: change: 6 (20.0%); p=0.2627 |
| Use of hypoglycaemic treatment (%) | CACS: Change at 3y: 5.6%  Controle: Change at 3y: 5.7%  DID: -0.06 (-4.6 to 4.5) |  |  |

Table 8 Changes in clinical risk factors. Values are median (25th, 75th percentile), n (%), mean +- SD change or 95% CI

|  | **Patrick G. O'Malley et al.** | | | **Jeffrey Lederman et.al.** | | | **Alan Rozanski et. Al.** | | | **Whitmore et al.** | | |
| --- | --- | --- | --- | --- | --- | --- | --- | --- | --- | --- | --- | --- |
|  | **CACS** | **Control** | **p** | **CACS** | **Control** | **p** | **CACS** | **Control** | **p** | **CACS** | **Control** | **Difference in change** |
| **Systolic blood pressure** (mmHg) | 0.96  +-0.80 | 1.65  +-0.79 | 0.54 | -1.23  +-16.03 | -5.93  +-12.43 | >0.05 | **-7 (-18,3)** | **-5 (-16, 6)** | 0.02 | -4.6 (-6.5, -2.8) | -3.5 (-5.4, -1.6) | -1.2 (-3.8, 1.5) |
| Diastolic blood pressure (mmHg) | 2.3  +-0.3 | 0.90  +-0.28 | 0.67 | -0.12  +-0.77 | -0.33  +-10.07 | >0.05 | -5 (-12,3) | -4 (-12, 4) | 0.50 | -1.0 (-2.5, 0.4) | -0.1 (-1.6, 1.3) | -0.9 (-2.9, 1.1) |
| Body mass index (kg/m2) | 0.38  +-0.12 | 0.35  +-0.11 | 0.84 | 0.10  +-1.33 | -0.51  +-3.32 | >0.05 |  |  |  | 0.3 (-0.1, 0.6) | 0.2 (-0.2, 0.6) | 0.1 (-0.4, 0.6) |
| **Waist circumference** (inches) |  |  |  |  |  |  | **0 (-3, 2)** | **1 (-2, 3)** | 0.01 | -0.9 (-1.8, -0.1) | -0.8 (-1.5, 0.02) | -0.1 (-1.2, 1.4) |
| Weight (kg) |  |  |  |  |  |  | 0 (-6, 7) | 1 (-5, 8) | 0.07 |  |  |  |
| Fasting Glucose (mg/dl) | 1.19  +-0.76 | 0.27  +-1.30 | 0.53 |  |  |  | 0 (-8, 7) | -2 (-8, 6) | 0.16 | 0.2 (-0.1, 0.4) | 0.2 (-0.01, 0.5) | -0.1 (-0.4, 0.3) |
| HbA1c (%) | -0.15  +-0.03 | -0.21  +-0.05 | 0.25 | 0.19  +-0.57 | 0.10  +-1.50 | >0.05 |  |  |  |  |  |  |

Table 9 Changes in follow-up lipid results. In the trial by Muhlestein et al. there was no significant difference in absolute change or percentag change but there was a significant difference in the mean total cholesterol at FUP CACS: 178.1±41.2 (173.5), Control: 167.2±39.1 (170.0) with p=0.0

|  | **O'Malley et al.** | | | **Lederman et.al.** | | | **Rozanski et.al.** | | | **Whitmore et.al.** | | | | **Muhlestein et.al.** | | |
| --- | --- | --- | --- | --- | --- | --- | --- | --- | --- | --- | --- | --- | --- | --- | --- | --- |
|  | **CACS** | **Control** | **p** | **CACS** | **Control** | **p** | **CACS** | **Control** | **p** | **CACS** | **Control** | **Adjusted difference^[[5]](#footnote-5)^** | **p** | **CACS** | **Control** | **p** |
| Total cholesterol (mg/dl) |  |  |  | **-3.35**  **+-26.56** | **-20.27**  **+-34.89** | <0.05 | -21  (49, 6) | -16  (44, 7) | 0.08 | -54.1  (-58,1, 46.4) | -3.9  (-7.7, 0.4) | -50.3  (-54.1, -42.5) | <0.001 | Absolute  -27.1+-36.8  Percentage  -13.1+-18.8 | Absolute  -19.9 +-36.9  Percentage  -8.8 +-16.7 | Absolute  0.11  percentage  0.06 |
| Low-density lipoprotein (mg/dl) | -6.38  +-1.51 | -5.77  +-1.94 | 0.81 | **1.15**  **+- 26.80** | **-17.43**  **+- 29.18** | <0.05 | **-17**  **(44, 7)** | **-11**  **(-41, 10)** | 0.04 | -46.4  (-54.1, 42.5) | -1.6  (-7.7, 3.9) | -46.4  (-54.1, -38.7) | <0.001 | Absolute  -23.8 +-34.7  Percentage  -14.7 +-41.8 | Absolute  -17.0 +-34.7  Percentage  -11.4 +-25.3 | Absolute  0.08  percentage  0.04 |
| High-density lipoprotein (mg/dl) |  |  |  | -2.77  +-7.62 | 1.13  +-7.96 | >0.05 | -1  (-6, 5) | -1  (-7, 5) | 0.28 | -1.6  (-3.9, 0.0) | 1.6  (-3.9, 0.0) | 0.15  (-3.9, 3.9) | 0.94 | Mean  1.4 +-7.0  Percentage  3.9 +-16.4 | Mean  -1.3 +-10.3  Percentage  -0.8 +-14.8 | mean:  0.03  percentage  0.05 |
| Triglycerides (mg/dl) |  |  |  | -11.62 +-45.43 | -23.73  +-54.26 | >0.05 | -10  (-42, 14) | -9  (-37, 14) | 0.40 | -7.7  (-11.6, -3.9) | -3.5  (-3.9, 7.7) | -7.7  (-15.5, -3.9) | 0.003 | Mean  -14.9 +-51.7  Percentage  -7.0 +-37.3 | Mean  -9.6 +-51.6  Percentage1.3 +-36.5 | Mean  0.04  percentage  0.02 |

1. In the trial by O`Malley et al Physical activity was measured using the validated Baecke Physical Activity questionnaire. Number represents a sports index ranging from 0 to 5. In the trial by Venkataraman it was measured in % of total patients evaluated. [↑](#footnote-ref-1)
2. In the trial by Denissen et al. the number of patients who consulted their GP was counted, with the most common stated reason being the wish to reduce CVD risk. [↑](#footnote-ref-2)
3. In the trial by O`Malley et al. motivation to change was based on a visual ladder score from 1 to 10 to measure overall behavioral stage of change [↑](#footnote-ref-3)
4. Stress was measured by the number and severity of responses to measures of 9 different domains of life (work, finances, relationships, caregiving burden, body image, sexuality, psychological support, health, and traumatic life experiences [↑](#footnote-ref-4)
5. Adjusted for sex, age and diabetes [↑](#footnote-ref-5)
