# Supplementary material for: Coronary atherosclerosis screening in asymptomatic adults using coronary artery calcium for cardiovascular prevention: a systematic review of randomised controlled trials and prospective cohorts
Source: BMJ Open. 2025 Jul 5;15(7):e101472. doi: 10.1136/bmjopen-2025-101472 (PMC12228433; doi:10.1136/bmjopen-2025-101472)
Supplement: Supplementary file 2 [file bmjopen-2025-101472.reviewer_comments.pdf]

PEER REVIEW HISTORY

BMJ Open publishes all reviews undertaken for accepted manuscripts. Reviewers are asked to complete a checklist review form and are provided with free text boxes to elaborate on their assessment. These free text comments are reproduced below.

ARTICLE DETAILS

Title (Provisional)

Coronary atherosclerosis screening in asymptomatic adults using coronary artery calcium for cardiovascular prevention: a systematic review of randomized controlled trials and prospective cohorts

Authors

Scheu, Victor; Alwan, Louhai; Gräni, Christoph; Gencer, Baris; Rodondi, Nicolas; Blum, Manuel R.

VERSION 1 - REVIEW

**Reviewer**	**1**
**Name**	**Teh, Andrew**
**Affiliation**	**Austin Health**
**Date**	**27-Mar-2025**
**COI**	**None**

With the use of coronary calcium scoring becoming increasingly prevalent, based on guidelines suggesting its use for restratification of patients with intermediate cardiovascular risk, the question posed by the authors is a valid and important one, given the relative lack of clinical outcome data for these recommendations. The key clinical questions posed for any screening tool include its accuracy in predicting outcomes and whether intervention based on a positive result can improve outcomes. Cost/benefit analysis is also then factored into the equation. As yet, there appear to be no compelling data to answer these questions to recommend widespread use of CACS to reduce CV morbidity/mortality in a primary prevention setting.

This article provides a systemic review and meta-analysis on the evidence for primary prevention screening for atherosclerosis using Coronary Artery Calcium scoring (CACS). The data set includes 7 RCTs and 1 observational study, with a total population of 51,544 patients. Not all studies included an interventional component. One study reported a very small, clinically insignificant reduction in BP control (131mmHg control vs 130mmHg CACS group). Several studies reported improved lipid profile. The results on use of cardiovascular medications was incongruent amongst studies, with some reporting an increase in statin utilisation and others showing a decrease in CV medication use. One study reported an increased adherence to statin medications. Although several studies reported on changes in

behaviour, only one reported improved motivation to lifestyle modification and one reported increased self-reported physical activity. Three studies reported on cardiovascular events and all-cause mortality for a total of 6,552 pts, with no significant impact on these outcomes. The authors correctly highlight the potential downsides of CACS screening including increased anxiety, referral for invasive testing, healthcare utilisation and false reassurance.

Overall there was a high risk of bias and substantial heterogeneity in the reported studies, which is a major limitation to the data analysis. In clinical practice, although the CACS is most strongly recommended for use in intermediate risk populations, it is commonly used outside of these guidelines. Consequently, the baseline CV risk varied greatly between studies in this analysis with a high degree of variability in CACS scores. For example, the authors cite one RCT including very low risk patients with a 10-year risk of <6% and 85% without any detectable CAC.

The conclusions do not appear adequately justified by the findings of the study. Only one study reported a clinically insignificant improvement in BP control. The findings with respect to improved lipid control were perhaps more consistent across 3 RCTs, although one showed deterioration and another no difference. The findings with respect to CV medication adherence were incongruent and only one study reported on improved motivation to change lifestyle. The final sentence in the conclusion appears to be the appropriate summary of the results identified.

Even if the primary finding of favourable effects on cardiovascular risk factor control are valid, given the very low event rates and highly variable baseline risk of the individuals included, the clinical benefits of CACS in asymptomatic individuals is highly questionable. Improving CV risk factor control in a patient already at low risk would seem to be a waste of valuable healthcare resources. Furthermore, despite the high total number of patients included, due to all of the limitations in trying to compare the included studies, no significant impact on clinical outcomes could be commented upon. The key clinical question on whether CACS can improve outcomes has not been addressed by the results of this meta-analysis given its inherent limitations and therefore does not provide any clinically meaningful information in addition to that already published.

**Reviewer**	**2**
**Name**	**Marschner, Simone**
**Affiliation**	**The University of Sydney, Westmead Applied Research Centre**
**Date**	**16-Apr-2025**
**COI**	**None**

This is simply a descriptive review of the papers they found on CACS.

Be careful quoting the results from papers. For example:

Only Rozanski et al. demonstrated a significant improvement in blood pressure (BP) between intervention and control groups (n= 2137; CACS 130 mmHg [119, 142]; Control: 131 mmHg [121, 144]; p=0.03),

The above statement is for baseline and not about the change in the groups. Please review all numbers quoted.

VERSION 1 - AUTHOR RESPONSE

Reviewer: 1

"With the use of coronary calcium scoring becoming increasingly prevalent, based on guidelines suggesting its use for restratification of patients with intermediate cardiovascular risk, the question posed by the authors is a valid and important one, given the relative lack of clinical outcome data for these recommendations. The key clinical questions posed for any screening tool include its accuracy in predicting outcomes and whether intervention based on a positive result can improve outcomes. Cost/benefit analysis is also then factored into the equation. As yet, there appear to be no compelling data to answer these questions to recommend widespread use of CACS to reduce CV morbidity/mortality in a primary prevention setting.

This article provides a systemic review and meta-analysis on the evidence for primary prevention screening for atherosclerosis using Coronary Artery Calcium scoring (CACS). The data set includes 7 RCTs and 1 observational study, with a total population of 51,544 patients. Not all studies included an interventional component. One study reported a very small, clinically insignificant reduction in BP control (131mmHg control vs 130mmHg CACS group). Several studies reported improved lipid profile. The results on use of cardiovascular medications was incongruent amongst studies, with some reporting an increase in statin utilisation and others showing a decrease in CV medication use. One study reported an increased adherence to statin medications. Although several studies reported on changes in behaviour, only one reported improved motivation to lifestyle modification and one reported increased self-reported physical activity. Three studies reported on cardiovascular events and all-cause mortality for a total of 6,552 pts, with no significant impact on these outcomes. The authors correctly highlight the potential downsides of CACS screening including increased anxiety, referral for invasive testing, healthcare utilisation and false reassurance.

Overall there was a high risk of bias and substantial heterogeneity in the reported studies, which is a major limitation to the data analysis. In clinical practice, although the CACS is most strongly recommended for use in intermediate risk populations, it is commonly used outside of these guidelines. Consequently, the baseline CV risk varied greatly between studies in this analysis with a high degree of variability in CACS scores. For example, the authors cite one RCT including very low risk patients with a 10-year risk of <6% and 85% without any detectable CAC."

1. "The conclusions do not appear adequately justified by the findings of the study. Only one study reported a clinically insignificant improvement in BP control. The findings with respect to improved lipid control were perhaps more consistent across 3 RCTs, although one showed deterioration and another no difference. The findings with respect to CV medication adherence were incongruent and only one study reported on improved motivation to change lifestyle. The final sentence in the conclusion appears to be the appropriate summary of the results identified."

   Thank you for your comment. We agree that our original conclusion may have suggested a more positive interpretation than warranted. We have revised the conclusions to align with the final sentence referred to by the reviewer (abstract and page (p.) 11, lines 410-411 and p. 12, line 416). Our review showed that while several trials have found favorable effects of CACS

screening, these effects were inconsistent and strongly dependent on study design, population risk, and co-intervention protocols. We now clearly state that, although suggestive of potential benefit in select contexts, the current evidence is insufficient to support broad clinical conclusions (p. 3, lines 107-108; p. 11, lines 410-411 and p. 12, line 416). The revised conclusion better reflects the observed heterogeneity and limitations.

2. "Even if the primary finding of favourable effects on cardiovascular risk factor control are valid, given the very low event rates and highly variable baseline risk of the individuals included, the clinical benefits of CACS in asymptomatic individuals is highly questionable. Improving CV risk factor control in a patient already at low risk would seem to be a waste of valuable healthcare resources."

We acknowledge and agree that the evidence for the clinical utility of CACS screening in low-risk individuals is likely limited or even absent. Accordingly, given the findings of this review, the use of CACS for screening in low-risk populations cannot be recommended.
In contrast, studies including participants at intermediate or higher risk (e.g., Denissen et al., ref. 22; Rozanski et al., ref. 27; Whitmore et al., ref. 26) more frequently reported favorable effects on lipid profiles, medication use, or adherence. This might suggest that the presence of subclinical atherosclerosis may act as a behavioral motivator in individuals who already have some elevated risk, a hypothesis we state in our review (p. 9, lines 332-341).
However, we also emphasize that this observed association between risk level and intervention effect must be interpreted with caution. The more pronounced effects in these studies are likely attributable not only to the underlying risk profile, but also to protocol-based pharmacological interventions implemented in the CACS groups in some trials (such as predefined statin or antihypertensive initiation). Therefore, any apparent advantage in intermediate to high risk populations may also reflect the effect of an intervention strategy coupled with the results of the CACS and not only the disclosure of the CACS values. (discussed p. 10, lines 342-370).
Additionally to the discussion we now also clearly state in the conclusion the potential impact of the influence of risk population and co-intervention (p.11, lines 410-411 and p. 12, line 416).
These insights support the reviewer's point and align with current guideline recommendations to restrict use of CACS to decision-making thresholds (e.g., borderline-to-intermediate risk, class IIb recommendation in ESC/ACC/AHA guidelines).

3. "Furthermore, despite the high total number of patients included, due to all of the limitations in trying to compare the included studies, no significant impact on clinical outcomes could be commented upon. The key clinical question on whether CACS can improve outcomes has not been addressed by the results of this meta-analysis given its inherent limitations and therefore does not provide any clinically meaningful information in addition to that already published."

We agree that our review does not provide conclusive or positive evidence regarding hard clinical endpoints such as cardiovascular events or mortality. This reflects the current state of the literature: only two RCTs (Muhlestein et al., ref. 24; Rozanski et al., ref. 27) and one observational study (Chi et al., ref. 30) in our sample reported on such endpoints, and all had low event rates and limited follow-up duration. As a result, no definitive statement can be made regarding the outcome benefit from CACS screening in asymptomatic individuals. This might be interpreted as a negative result, but in fact it should be interpreted as limited evidence - in our opinion this is a highly relevant finding, given the fast-rising use of CACS for screening purposes despite insufficient evidence to support this use. Furthermore, we believe the review contributes meaningful insight beyond prior work through 1) focusing specifically on the effect of CACS screening (not prediction) on CV risk factor control, health behavior and clinical events; 2) synthesizing RCT evidence published since the last comprehensive review in 2014 (e.g., van der Aalst et al., ref. 25; Denissen et al., ref. 22; Whitmore et al., ref. 26;

Muhlestein et al., ref. 24); 3) highlighting the heterogeneity in intervention design and downstream effects—ranging from passive feedback of results to protocolized treatment escalation—thus informing future trial design and implementation frameworks.

Reviewer: 2

1. "This is simply a descriptive review of the papers they found on CACS."

   We thank the reviewer for this comment. We would like to clarify that our review is not a narrative review but a systematic review and we followed a systematic approach in accordance with the PRISMA guidelines, including a predefined and published search strategy, explicit inclusion and exclusion criteria, and structured data extraction. While the findings are primarily presented in a descriptive manner, this reflects the heterogeneity of study designs, populations, and outcomes among the included studies, which precluded meta-analysis or more formal synthesis methods.
   Nonetheless, as we have pointed out in answer to reviewer 1's comment #1, the revised conclusion better reflects the observed heterogeneity and limitations.

2. "Be careful quoting the results from papers. For example: Only Rozanski et al. demonstrated a significant improvement in blood pressure (BP) between intervention and control groups (n= 2137; CACS 130 mmHg [119, 142]; Control: 131 mmHg [121, 144]; p=0.03), The above statement is for baseline and not about the change in the groups. Please review all numbers quoted."

   Thank you for your comment. Upon review, we acknowledge that we inadvertently only cited the baseline blood pressure values from Rozanski et al. and forgot to add the changes over time.
   The additional data from the study are as follows: Change in systolic BP (mean, mm Hg): CACS group: –5 mm Hg (IQR –16 to –6), Control group: –7 mm Hg (IQR –18 to –3), with a p-value of 0.02, indicating a statistically significant difference in favor of the intervention.
   We have corrected this in the manuscript. In addition, we have re-reviewed all other numerical data quoted from the included studies to ensure accuracy and consistency throughout the manuscript and made corrections or additional explanations where necessary (p. 2, lines 52-54; p. 7 lines 254-258 and p. 7 line 264).
